# Community-acquired pneumonia mortality trends according to age and gender: 2009 to 2019

**DOI:** 10.1186/s12890-025-03875-8

**Published:** 2025-08-14

**Authors:** João Gonçalves-Pereira, Filipe Froes, Filipa Gonçalves Pereira, António Diniz, Henrique Oliveira, Paulo Mergulhão

**Affiliations:** 1 Emergency and Intensive Care Medicine Department, Hospital Vila Franca de Xira, Vila Franca de Xira, Portugal; 2 Department of Intensive Care Medicine, Faculty of Medicine, Lisbon University, Lisbon, Portugal; 3 Grupo de Infeção e Desenvolvimento em Sépsis, Porto, Portugal; 4 Chest Department, Hospital Pulido Valente - Centro Hospitalar Universitário Lisboa Norte, Lisboa, Portugal; 5 Pulmonology Consultant, Carcavelos, Portugal; 6 Center for Mathematical Analysis, Geometry and Dynamical Systems (CAMGSD), Instituto Superior Técnico, University of Lisbon, Lisbon, Portugal; 7 Intensive Care Medicine Department, Hospital Lusíadas Porto, Porto, Portugal; 8 Department of Medicine, Faculty of Medicine, University of Porto, Portuguese Intensive Care Society, Porto, Portugal; 9 Intensive Care Medicine Department, Hospital Vila Franca de Xira, Estrada Carlos Lima Costa, N2, Vila Franca de Xira, 2600-009 Portugal

**Keywords:** Community-acquired pneumonia, Hospital mortality, Portugal, Season, Age groups

## Abstract

**Background:**

Community-acquired pneumonia (CAP) is one of the leading causes of morbidity and mortality worldwide. It remains unknown whether recently introduced prevention strategies, such as vaccination, may have impacted its mortality. Moreover, it is important to understand whether these trends are similar for all patients or if some groups may have different outcomes.

**Methods:**

We evaluated the CAP hospitalization data for adult patients, from the Portuguese mainland Hospital diagnosis database. We included all patients discharged with CAP between 2010 and 2019. We assessed Hospital mortality for the whole population and according to age and gender. We also examined the monthly number of admissions and mortality.

**Results:**

We identified 462,910 CAP admissions, of which 54% were male (mean age 76.8 ± 14.5years). We divided our population into 4 age groups: 18-40years (3.2% of episodes); 41-65years (14.9%); 66-80years (31.6%); >80 years (50.2%). Mortality increased sharply with age, to more than 40% in older patients, highlighting the disproportionate burden of respiratory diseases in this age group. Although CAP mortality remained stable until 2016 (roughly 23%), a slight decreasing trend was noted afterwards (from 23.1 to 21.8%). In hospitalized patients, the relative risk of dying with CAP, consistently decreased during this period, from odds ratio 5.5, 95% CI 5.4–5.7 to odds ratio 4.3, 95% CI 4.2–4.4. As much as 25% of deaths related to CAP occurred during the first 48 h after hospital admission. While CAP admissions peaked in January, mortality was higher during the summertime.

**Conclusions:**

Community-acquired pneumonia remains associated with a high risk of death. Mortality increased with age from 40 years onwards. However, the relative risk of dying with CAP in the Hospital decreased during this decade.

**Graphical Abstract:**

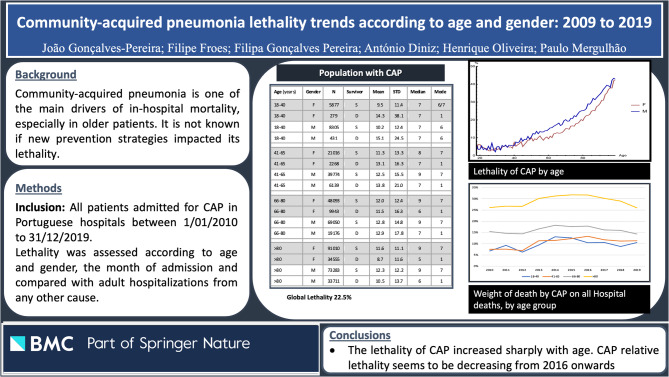

**Supplementary Information:**

The online version contains supplementary material available at 10.1186/s12890-025-03875-8.

## Background

Community-acquired pneumonia (CAP) remains a common cause of infection and Hospital admission, with a high burden of morbidity and mortality, although significant regional variations are usually noted [1].

Despite the advances in CAP treatment and prevention (mostly related to vaccines against Influenza virus and invasive pneumococcal disease), at least 17% of patients with CAP still require hospitalization [2], and those with non-resolving CAP may experience a more than 5 -fold increase in the risk of death [3].

There are considerable differences in the characteristics of patients hospitalized with CAP, especially regarding the prevalence of comorbidities that may increase the incidence of CAP, such as chronic obstructive pulmonary disease (COPD) [4]. However, age remains the main factor associated with CAP incidence and mortality [5–7].

Accordingly, older patients have CAP more often, which may translate into a prolonged Hospital length of stay, high bacteremia incidence, and mortality [7, 8]. Risk factors for CAP, prevention strategies, and therapeutic priorities of older patients may differ from those of the young, which should inform clinical practice. Escalating the level of care [9] and increasing efforts to identify CAP etiology [10] have been proposed to help decrease mortality for this group.

In this study, we address the incidence and mortality of CAP and their trends for 10 years, for different groups of patients, according to gender and age group, to evaluate their differences throughout this period and to provide data to guide prevention and treatment strategies.

## Methods

### Data retrieving

The Central Administration of the Health System of the Portuguese Ministry of Health records administrative and clinical discharge data for all admissions to National Health System Hospitals, covering almost the whole resident population of mainland Portugal. This database consists of an anonymized database that includes all discharge diagnoses of Hospital inpatients, either alive or deceased, introduced by specially trained medical staff. Supplementary File 1 describes in detail the ICD-9 and ICD-10 codes used to identify the different pathologies for this study.

This study evaluated all Hospital adult admissions (> 18 years) of patients admitted between 1-1-2010 and 31-12-2019.

We computed administrative data, namely age and gender, and the presence of chronic comorbidities.

Mortality at Hospital discharge and Hospital length of stay (LOS) were the primary outcomes evaluated.

The Central Administration of the Portuguese National Health System approved this study., before giving access to the data, according to its Privacy and Personal Data Protection Policy (available at https://www.acss.min-saude.pt/2023/06/05/politica-de-privacidade-e-de-protecao-de-dados-pessoais/). Since all protected individual patient information was inaccessible, and only aggregated data was available, the requirement for patient informed consent was waived.

The study was performed in agreement with the Declaration of Helsinki statement of ethical principles for medical research.

### Community-acquired pneumonia

Patients with a primary diagnosis of CAP were segregated for further analysis. Patients under 18 years of age, those for whom pneumonia was not the main diagnosis, immunocompromised patients, and those who did not have pneumonia “present on admission” were excluded.

We divided our population into four groups based on age at Hospital admission: 18–40, 41–65, 66–80, and > 80. We also calculated the incidence and mortality of CAP according to the month of admission to the Hospital.

We adopted a descriptive statistical analysis approach to obtain the relevant indicators for this study. We computed rates of CAP per 1000 hospitalization episodes (with 95% confidence intervals) according to age, gender, month, and year of admission. We also calculated the evolution of the absolute number of CAP episodes and the trends in admission rates and mortality over 10 years for the different subgroups. Finally, we calculated the odds ratio (OR) for mortality, with 95% confidence intervals (CI).

Absolute CAP mortality was calculated and compared to the whole Portuguese population.

The Hospital LOS was computed for patients with CAP, according to age and gender, and compared with patients without CAP.

### Statistical analysis

Continuous variables were expressed as mean (standard deviation) and/or median and/or mode according to data distribution; the discrete variables were expressed as total number (percentage). The statistical significance analysis was performed using the Chi-square test (for discrete variables), Student’s T, Mann Whitney, or Kolmogorov Smirnov tests (for continuous variables).

All the calculations presented were obtained using the statistical software package STATA (release 11; StataCorp, College Station, TX, USA), the Wolfram Mathematica 13.3.1 (Champaign, IL, USA), and the Microsoft Excel spreadsheet (Microsoft Corp., Redmond, WA, USA).

## Results

### Incidence and mortality

During the 10-year period under study, there were 462,910 CAP admissions to the Portuguese mainland Hospitals (54% males), corresponding roughly to 5 episodes/1000 population per year. The mean age was 76.8 ± 14.5 years, being slightly lower for males (74.7 ± 14.6 vs. 79.1 ± 14.1 years, *p* < 0.001). More than 50% of CAP patients were > 80 years (Table 1). Although the median time in the hospital was similar in all groups (roughly 6–8 days), the mode was much lower in patients who died, unveiling that most deaths (25%) occurred during the first 48 h after hospital admission.

**Table 1:** Hospital length of stay according to age, and gender for community-acquired pneumonia survivors and deceased

Age (years)	Sex	Number	Mean	STD	Median	Mode
**Survivors**						
18-40	F	5877	9.45	11.44	7	6/7
	M	8305	10.20	12.42	7	6
41-65	F	21016	11.27	13.27	8	7
	M	39774	12.49	15.48	9	7
66-80	F	48093	12.02	12.43	9	7
	M	69050	12.76	14.80	9	7
>80	F	91010	11.63	11.09	9	7
	M	73283	12.27	12.18	9	7
**Deceased**						
18-40	F	279	14.28	38.06	7	1
	M	431	15.11	24.48	7	6
41-65	F	2268	13.07	16.27	7	1
	M	6139	13.79	21.00	7	1
66-80	F	9943	11.53	16.31	6	1
	M	19176	12.93	17.67	7	1
>80	F	34555	8.70	11.64	5	1
	M	33711	10.46	13.73	6	1

*F* Female, *M* Male, *S* Survivors, *D* Deceased, *STD* Standard deviation

**Fig. 1:**
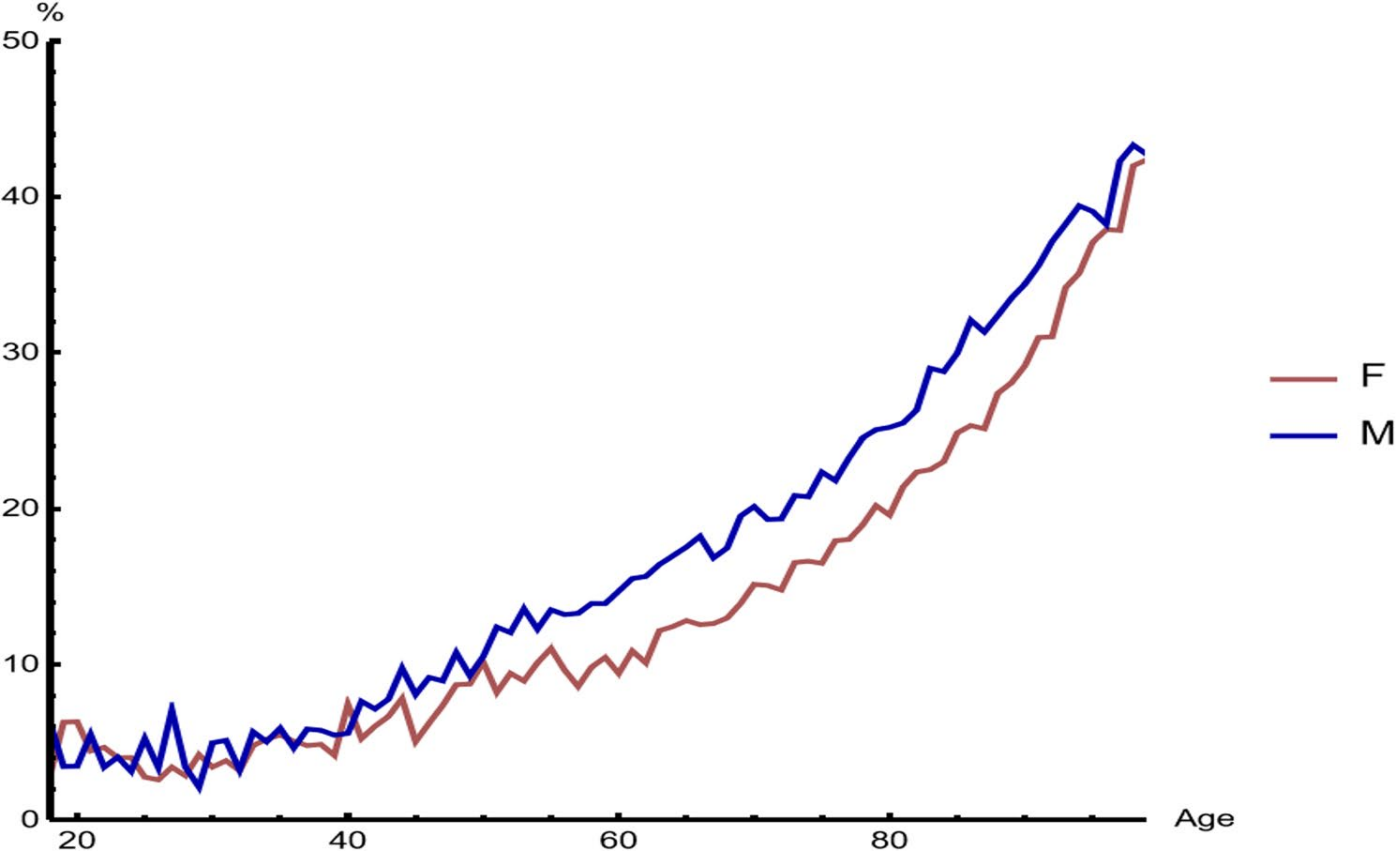
Mortality of community-acquired pneumonia according to age and gender

The global mortality rate during these 10 years was 22.5%. Non-survivors were older (age 81.6 ± 11.0 vs. 75.3 ± 15.1, *p* < 0.001). There was a strong relationship between mortality and age. Accordingly, from 40 years onwards (Fig. 1), mortality increased sharply, from roughly 5% to over 40%. This corresponded to a progressive increase in the mortality risk, OR 8.33, 95% CI 7.7-9.0 for the population > 80 years, compared with those 18–40 years (Table 2).

**Table 2:** Community-acquired pneumonia mortality risk according to age and gender

Age group	N	Percent of CAP episodes	Mortality	Age (OR, 95%CI)	Male vs. female (OR, 95% CI)
18-40	14,892	3.2%	4.78%	Control	1.19 (1.17-1.22)
41-65	69,197	14.9%	12.3%	2.80 (2.59-3.03)	1.05 (1.02-1.07)
66-80	146,262	31.6%	17.9%	4.37 (4.05-4.71)	1.42 (1.35-1.49)
>80	232,559	50.2%	29.5%	8.33 (7.72-8.99)	1.10 (0.94-1.49)

*CAP* community-acquired pneumonia, OR (95% CI)– Odds Ratio with 95% confidence interval

Globally, there were no significant differences between genders (males 22.6% vs. females 22.4%, *p* = 0.111). However, in each of the age sub-groups, males had a significantly higher mortality compared with females (Table 3). This can be explained by the significantly larger absolute number of females in the sub-group > 80 years, with the highest mortality.

**Table 3:** Impact of community-acquired pneumonia on the overall mortality of all adult admissions

	Total of admissions	Global mortality	CAP	Death with CAP*	OR** (95% CI)
18-40	1,574,747	0.5%	14,892 (0.9%)	9.5%	10.4 (9.6-11.2)
41-65	2,283,731	3.6%	69,197 (3.0%)	10.4%	3.7 (3.6-3.8)
66-80	2,119,799	7.7%	146,262 (6,9%)	16.1%	2.5 (2.4-2.5)
>80	1,453,297	16.3%	232,559 (16.0%)	29.0%	1.9 (1.8-1.9)

*CAP* community-acquired pneumonia, OR (95% CI)– Odds Ratio with 95% confidence interval

*Percentage of all patients admitted to the Hospital with community-acquired pneumonia among those who died in the Hospital

** Odds Ratio for dying if hospitalized with community-acquired pneumonia compared with all other causes

Although the absolute mortality of CAP increases with age, older patients often have other causes of death. Consequently, in this age group, the relative risk of dying from CAP (among all causes of death) is lower than that of younger populations. Cardiovascular causes of death, which are common in older patients, are rare among younger individuals, and only 0.5% of young patients admitted to the hospital die. Therefore, while absolute mortality from CAP is lower younger patients, CAP remains one of their leading causes of death. As a result, the relative risk of dying from CAP among those who die is higher in this group.

Patients with CAP had a median Hospital LOS of 8 days, lower than the mean, unveiling prolonged hospitalizations for some patients. The mode of non-survivors was 1 day, reinforcing the importance of a sepsis fast-track approach to these patients [11]. The median Hospital LOS was similar between genders. Of note, deceased younger patients, either male or female, have a significantly higher mean LOS than survivors, whilst deceased older patients were in the Hospital less time than survivors (Table 1).

### Weight of Community-acquired Pneumonia on the death risk of total admissions

During the study period, there were a total of 7,431,574 Hospital adult admissions, including 6.2% admitted for CAP. The all-cause mortality was 6.6%. Patients with CAP had a disproportionately high mortality, OR 5.14, 95% CI 5.10–5.18. This very high relative mortality risk decreased with advancing age in patients with CAP (Table 3).

**Fig. 2:**
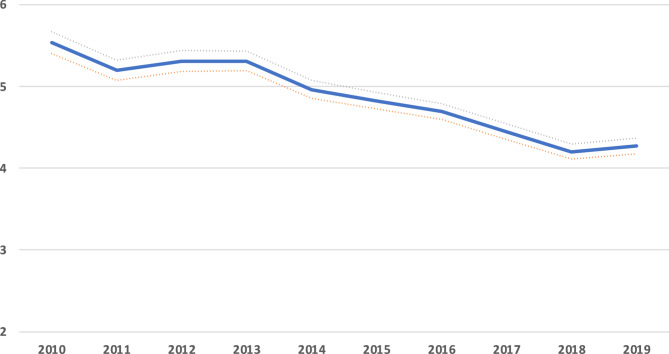
Odds Ratio (with 95% confidence interval) for dying in the Hospital with community-acquired pneumonia among deceased patients

**Fig. 3:**
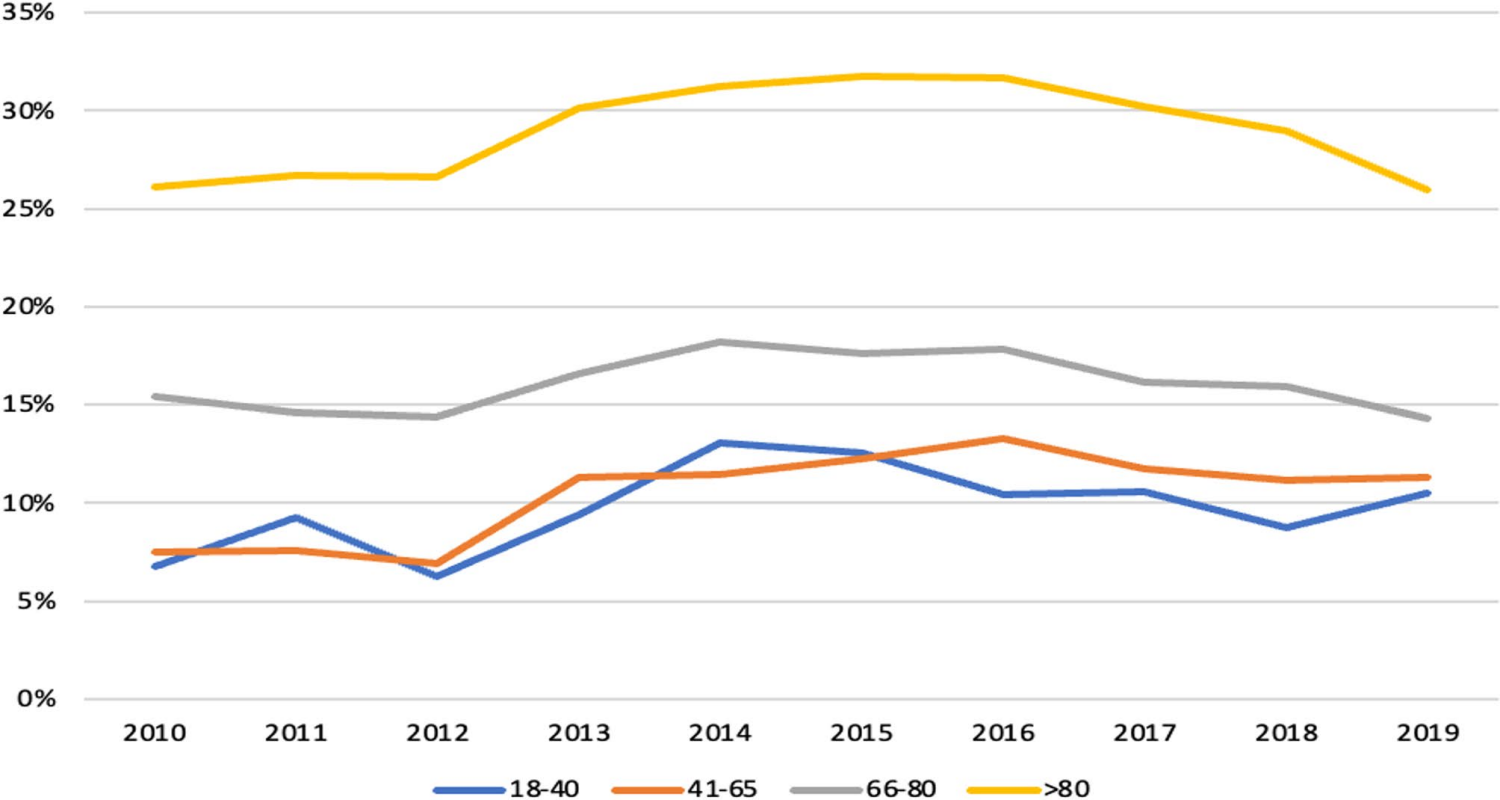
Hospital community-acquired pneumonia relative mortality (compared to all other adult hospitalizations mortality), per year and age group

From 2014 onwards, there was a marked decrease in the weight of CAP in in-Hospital mortality (Fig. 2), particularly in the older population (Fig. 3).

### Trends in mortality

**Fig. 4:**
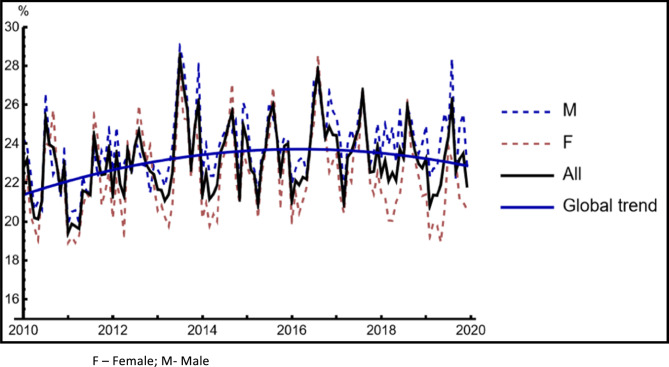
Monthly mortality of patients with community-acquired pneumonia admitted to the Hospital

Mortality increased during the first years of the decade (Fig. 4), but that was followed by a decreasing trend, noted from 2016 onwards.

There were differences in mortality trends, reported in Table 4. The group aged 41–65 years presented a significant increase in mortality, which was not observed in the other groups. Notably, males aged 66–80 years and females > 80 years experienced a decrease in mortality.

**Table 4:** Trends in community-acquired pneumonia hospital mortality for 10 years according to age and gender

Age (years)	Slope m	Basis b (%)	Trend
>80 M	0.00173	31.35	Increasing
>80 F	-0.01970	29.04	Decreasing
66-80 M	-0.03521	20.40	Decreasing
66-80 F	0.00585	17.22	Increasing
41-65 M	0.03237	11.49	Increasing
41-65 F	0.02698	8.27	Increasing
18-40 M	0.00251	4.82	Increasing
18-40 F	0.01122	3.89	Increasing

Mortality=m x +b. *F* Female, *M* Male

In the younger population, a convergence of the mortality between genders was noted, and the mortality of females surpassed even the mortality of males in 2019 (5.46% vs. 3.96%, respectively), a difference that was not significant (*p* = 0.96). Overall, there were more episodes of CAP in males but no significant differences in mortality were noted (OR 1.01, 95% CI 1.00-1.03).

**Fig. 5:**
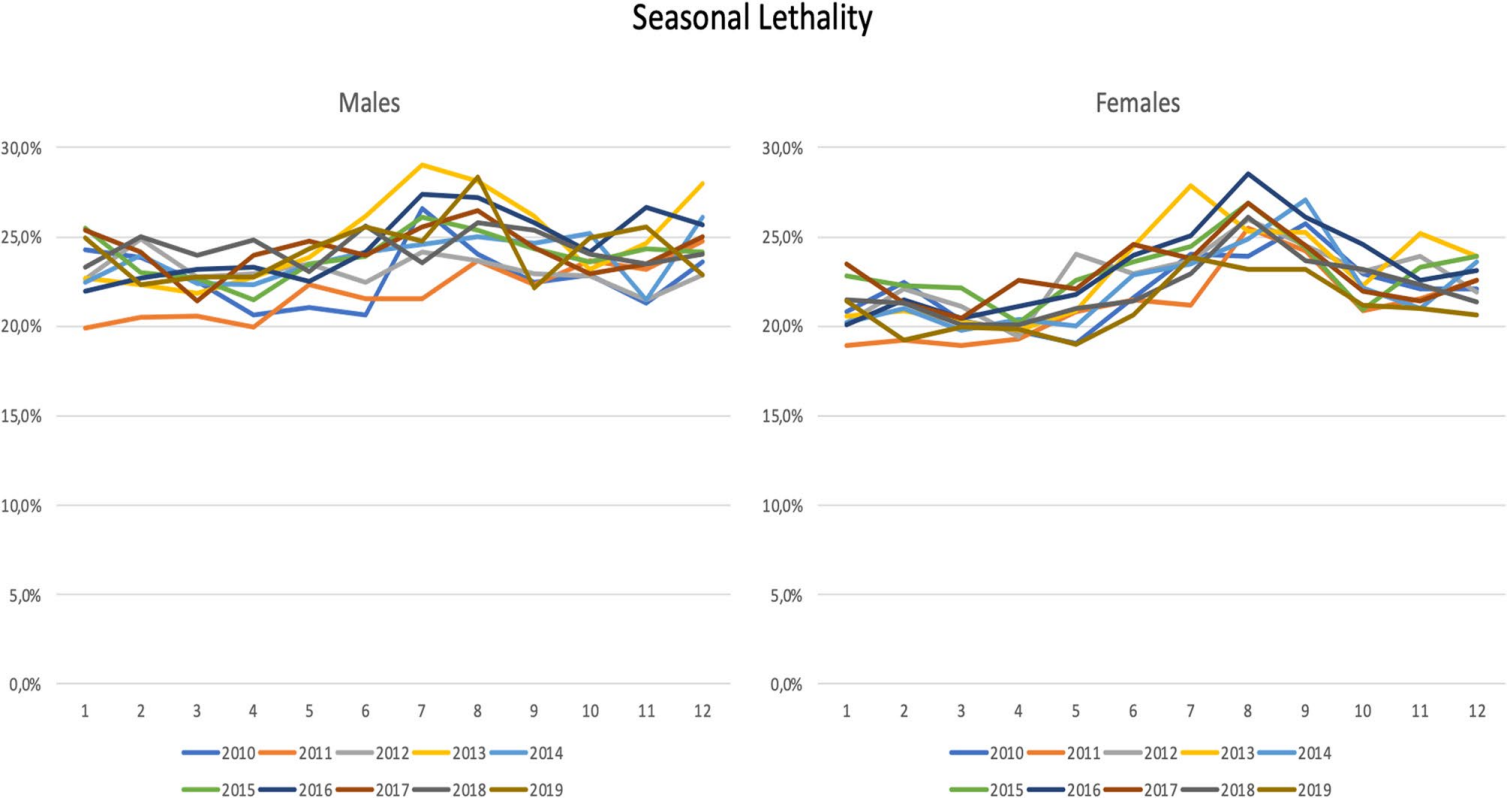
Community-Acquired Pneumonia Hospital Mortality per month

As expected, admissions peaked during the Wintertime. Admissions for CAP during January were more than double of the admissions during August. However, curiously, a peak of mortality (especially in women) was noted in Summer, mainly from July to September (Fig. 5). This was evident for all age groups.

## Discussion

In this study, we present the hospital mortality of a large population admitted during a 10-year period to the Portuguese mainland Hospitals. We identified 462,910 patients, with a mean mortality of 22.5%. This rate increased sharply with age, from 40 years onwards, reaching over 40% (Fig. 1). No significant differences were noted between genders. CAP accounted for 6.2% of all hospitalizations and was the main diagnosis in more than 1/5 (21.8%) of the deaths occurring in the Hospital. The relative weight of CAP mortality on Hospital mortality decreased during this period (Fig. 3), particularly in the older population.

Cold weather seems to be associated with an increased number of CAP admissions to the Hospital. However, Hospital CAP mortality peaked during the Summertime for all age groups (Fig. 5).

A recently published Portuguese study reported a progressive increase in mortality until 2014 [12]. We note that this increase was mainly due to an increase in admissions of older patients (> 80 years) with higher mortality. Interestingly, this mortality seems to be decreasing, especially from 2016 onwards. Consequently, we think that the evaluation of trends in CAP mortality should always consider age. However, this should be interpreted with caution, as these findings were based solely on observational data, the differences were minimal, and the rates tend to fluctuate over time.

Despite these improvements in incidence and mortality, the rates of CAP hospitalization remain very high, particularly among older patients, ranging from 847 to 3500 per 100,000 persons-year, according to the used criteria [13]. In our study, 6.2% of all adult patients admitted to the Hospital had CAP and an increased relative risk of dying, which decreased from OR 5.5, 95% CI 5.4–5.7 to OR 4.3, 95% CI 4.2–4.4 (Fig. 2) during these 10 years. Cardiovascular events may contribute to this, especially in older patients with pre-existing coronary disease, and their occurrence was noted to be associated with a significant increase in mortality risk ranging between 1.39 and 5.49 [14]. The significant burden of CAP and its poorer clinical outcomes have also been observed in critically ill patients, with higher in-hospital mortality and readmission rates, and greater healthcare resource utilization, even after adjusting for comorbidities and socioeconomic variables [15].

Despite this high burden of incidence and mortality, which could be over 4 times higher than that of myocardial infarction or stroke, the cost of preventive strategies for CAP was less than 10% of the investment allocated to the prevention of these other diseases [16].

We believe that more efforts, namely tailored strategies, should be made to prevent CAP in older patients and other high-risk groups. In our study, 50.2% of patients who died with CAP were over 80 years old, and even higher rates (66.8%) have been reported [17].

Swallowing dysfunction evaluation and prevention may help prevent microaspiration and severe CAP [18, 19]. Improving nutrition, identifying and decreasing the progression of frailty and avoiding polypharmacy [20], may all help decrease CAP in this at-risk group [9]. According to our data, a reduction in Hospital admissions of these patients of even 5% may translate into a reduction of more than 130,000 patient days per year, with substantial health cost benefits. A public health policy addressing CAP prevention, especially reinforcement of an active vaccination policy (with community interventions) and the promotion of active ageing, should be promoted to improve health indicators.

Another interesting finding of our study is the seasonal incidence of CAP, with a significantly higher risk of admission during the Wintertime, particularly in January [21, 22]. This may allow tailoring or resource allocation to address this predictable increase in CAP incidence. Moreover, the striking differences in incidence and mortality of patients with CAP according to age group should foster different prevention and treatment policies directed at their different risk factors and medical needs. Finally, the peak of CAP mortality in summer, possibly related to a higher prevalence of bacterial disease other than *Streptococcus pneumoniae* and a residual prevalence of viral infection [23, 24], should be considered when addressing these patients in the emergency department. Complications related to heat and delays in recognizing CAP (especially when patients do not present with classical symptoms) may also be associated with this mortality peak. Besides, the temperature-attributable mortality may shift from winter to summertime, driven by the very large decrease in the risk of death due to cold temperatures [25].

In 2015, the Portuguese Health authorities recommended the 13-valent and the 23-valent pneumococcal conjugate vaccines to all patients older than 65 years [26]. This, alongside a sustained increase in Influenza Virus vaccination coverage rates (over 70% in patients > 65 years [27], may have contributed to the decrease in CAP mortality that we unveiled. The benefit of anti-pneumococcal vaccination for the prevention of severe CAP is well known [28], and benefits to the older population may also be derived from pediatric vaccination [29], introduced in the National Immunization Plan in 2015. A very large immunization rate (over 95%) was noted almost immediately [30]. We believe that this might have had unexpected beneficial collateral effects, as ill children may be more prone to be infected by viral agents and pass them to older relatives. This could contribute to the relative decrease in CAP mortality that we found, which surpassed a potential decrease in Pneumococcal incidence. Besides, this improvement in prognosis may reflect broader improvements in clinical management of CAP over time [31], namely, early use of combination antibiotic therapy [32].

This study has some important limitations: Patients were selected from an administrative database that did not allow for the confirmation of the diagnosis of CAP, nor the stratification of patients according to comorbidities (namely cardiovascular disease, COPD or diabetes). Also, we were not able to provide stratification by comorbidities or functional status, which are known to be major CAP outcome modifiers. Besides, the study provided limited microbiological data. Third, all data come from Portugal only, and differences between health policies in different countries may influence the outcomes.

However, this study also has some strengths: We incorporated data from a large period (10 years) and evaluated a very large database, encompassing almost all hospitalized adult CAP patients, as all public Hospitals in mainland Portugal are required by law to send all their data to the Central Administration of the Health System.

Besides, only patients admitted before 2019 were included, to exclude a possible effect of the Coronavirus Disease 19 (COVID-19) pandemic.

## Conclusions

Community-acquired pneumonia (CAP) is a common cause of adult Hospital admission and is associated with an increased risk of death. This risk increased from the age of 40 onwards, reaching over 40% in older patients. CAP’s relative risk of mortality (when compared to the overall population) decreased between 2010 and 2019, especially for those aged 66–80. Striking differences in prevalence and mortality were noted for the different age groups, which should foster different healthcare policies.

## Supplementary Information

Supplementary Material 1.

## Data Availability

The datasets supporting this study’s findings are available from the Central Administration of the Portuguese National Health System. Still, restrictions apply to their availability, according to their Privacy and Personal Data Protection Policy. Access to raw data can be requested from Cláudia Borges (cborges@acss.min-saude.pt), Luís Faustino (lfaustino@acss.min-saude.pt), Vanessa Silva (vcsilva@acss.min-saude.pt). HO act as the guardian of the raw data used in this study. After permission from the Central Administration of the Portuguese National Health System, authors can provide the data used in this study, upon reasonable request.
