# Supplementary material for: Community-acquired pneumonia mortality trends according to age and gender: 2009 to 2019
Source: BMC Pulm Med. 2025 Aug 14;25:391. doi: 10.1186/s12890-025-03875-8 (PMC12351841; doi:10.1186/s12890-025-03875-8)
Supplement: Supplementary file 1 — Supplementary Material 1. [file 12890_2025_3875_MOESM1_ESM.docx]

**Supplementary File 1**

**ICD-9 and ICD-10 codes used in this study**

The diagnosis of pneumonia was identified using the following codes: ICD-9-CM codes 480 to 486 and 487.0, and ICD-10-CM codes J12 to J18, J10.0X, and J11.0X.

Starting from 2013 onwards, this coding was supplemented with the "present on admission" code, added to the pneumonia diagnosis. Individuals with HIV infection (ICD-9-CM codes: 042-044; ICD-10-CM codes: B20) were excluded from the study, as well as those who were immunocompromised due to anti-cancer or immunosuppressive treatment (ICD-9-CM code: E933.1; ICD-10-CM codes: V87.41, D61.810, D70.1), and transplant recipients (ICD-9-CM codes: V42, except for corneal transplant status V42.5; ICD-10-CM codes: T86.XX and Z94.X, except for corneal transplant status Z94.7).
